# Acalculous cholecystitis in patient with hepatic hypogenesis and chilaiditi sign

**DOI:** 10.11604/pamj.2015.21.297.7675

**Published:** 2015-08-24

**Authors:** Adriá Rosat, Daniel Eiroa

**Affiliations:** 1 Department of General Surgery, Hospital Universitario Nuestra Señora de Candelaria, Tenerife, Spain; 2 Radiodiagnostic Service, Hospital Universitario Nuestra Señora de Candelaria, Tenerife, Spain

**Keywords:** Acaculous Cholecystitis, hepatic hypogenesis, chilaiditi sign

## Image in medicine

A 66-year-old male patient was admitted in our outside hospital's emergency room after 4 days of epigastric pain, nausea and vomiting. No fever, dysthermia or respiratory symptoms were related. The pain irradiated to right hypochondria and even to right chest. Laboratory tests showed slight elevation of liver enzymes and leukocytosis. Abdominal CT revealed hepatodiaphragmatic colonic interposition, segmental agenesis of the right hepatic lobe (A) and a gallbladder with thickened walls and inflammatory changes without any stone (B). The patient did well with antibiotic treatment and was discharged home. Hepatodiaphragmatic interposition of the intestine, known as Chilaiditi sign or syndrome, was first described by Demetrius Chilaiditi in 1910, with an incidence of 0.025-0.28%. Predisposing factors include absence of the normal suspensory ligaments of the transverse colon, redundant colon, right hemidiaphragm elevation, atrophy or hypogenesis of the right hepatic lobe, etc. Agenesis or hypogenesis of the right liver lobe is an extremely rare congenital anomaly and one of the rare causes of Chilaiditi sign or syndrome. It is considered to be caused by a failure of the right portal vein to develop, or an error of mutual induction between the primitive diaphragm and the endodermal diverticulum representing the primitive liver. The differential diagnoses of Chilaiditi syndrome include pneumoperitoneum, diaphragmatic hernia, subdiaphragmatic abscess, bowel obstruction and volvulus. No chirurgic intervention is required for a patient with Chilaiditi sign, they respond to medical management, and surgery is reserved for those who do not respond to usual conservative line of management.

**Figure 1:**
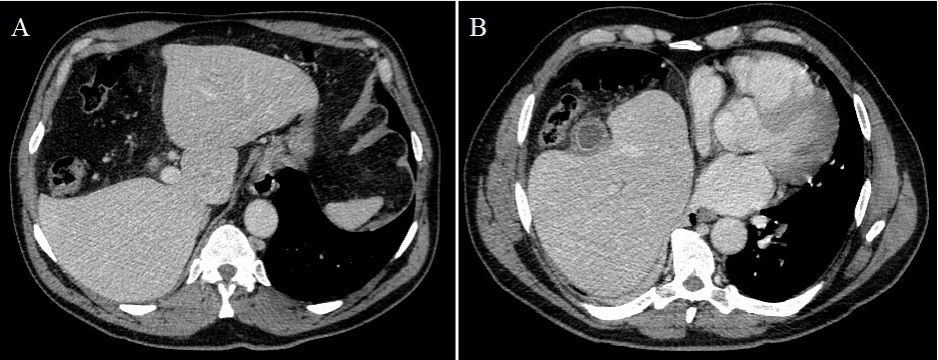
(A) colonic interposition between the liver and the diaphragm, in the place left by a segmental agenesis of the right hepatic lobe; (B) thickened walls gallbladder and inflammatory changes in surrounding fat without any stones nor bile duct dilatation

